# Antiseptic Ointment

**Published:** 1898-09

**Authors:** 


Antiseptic Ointment. ℞.—Iodoform, ʒj ; acid boracic et antipyrine, āā ʒv; petrolatum, ℥ vi. et ʒij. M.

The cancer germ, leydenia gemunipara sehaudin, is the name given to a parasite amœboid rhizopod, which Berlin professors have recently found in the fluid taken from patients suffering from cancer of the stomach, and which they think may possibly be the cause of the disease.

